# Optimisation of Laccase Activity From *Bacillus atrophaeus* Using Response Surface Methodology: A Proof‐Of‐Concept Dye Decolourisation Study

**DOI:** 10.1111/1758-2229.70138

**Published:** 2025-07-01

**Authors:** Kubra Kocak, Arzu Altin Yavuz, Suleyman Berberler, Cansu Filik Iscen

**Affiliations:** ^1^ Graduate School of Natural and Applied Sciences Eskisehir Osmangazi University Eskisehir Turkey; ^2^ Faculty of Sciences, Department of Statistics Eskisehir Osmangazi University Eskisehir Turkey; ^3^ Faculty of Education, Department of Mathematics and Science Education Eskisehir Osmangazi University Eskisehir Turkey

**Keywords:** azo dye degradation, *Bacillus atrophaeus*, bacterial laccase, dye decolourisation, optimisation, response surface methodology (RSM)

## Abstract

This study investigates the enhancement of laccase activity, a copper‐containing enzyme involved in oxidative biodegradation. The enzyme was studied in *Bacillus atrophaeus* , newly isolated from paper mill wastewater. Initial optimisation of key factors, including carbon and nitrogen sources, incubation time, inoculum size, pH, temperature, and CuSO4 concentration. Subsequently, a systematic refinement of selected parameters was performed through response surface methodology, a statistical optimisation technique. The maximum laccase activity of 0.057 U/mL was achieved under the following conditions: pH 8.0, 35.28°C, 1.5% CuSO_4_, 0.5% inoculum size, 3.7 g/L fructose and 1.08 g/L yeast extract. Under these conditions, a 2.51‐fold enhancement in enzymatic activity was achieved compared to pre‐optimised conditions. The optimised enzyme activity was then tested for its ability to decolourise dyes, specifically Congo red, burazol black and burazol navy. Congo red decolourisation exhibited a 2.95‐fold increase after 72 h under optimised conditions, whereas burazol black and burazol navy dyes remained unaffected. These findings underscore the potential of optimised laccase‐based methods for efficient dye wastewater treatment. Using response surface methodology, key parameters were fine‐tuned to enhance laccase activity and decolourisation efficiency, advancing sustainable bioremediation in environmental biotechnology.

## Graphical abstract

Summarising the optimisation of laccase production from *Bacillus atrophaeus* using response surface methodology (RSM) and its application in dye decolourisation. Created with BioRender.com.

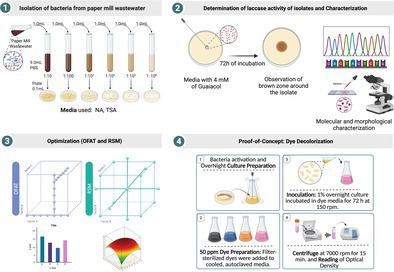

## 1 Introduction

Laccase enzymes (oxygen oxidoreductases; EC 1.10.3.2), part of the phenol‐oxidases group and the blue‐copper protein family, catalyse the oxidation of a wide variety of phenolic and non‐phenolic substrates by reducing molecular oxygen to water (Johannes and Majcherczyk 2000; Altıntas 2011; Othman and Flaifil 2025). Their broad substrate specifity, coupled with high catalytic efficiency, makes them valuable biocatalysts in several industrial applications including bioremediation, textile processing, pulp and paper bleaching and wastewater treatment (Rodriguez‐Couto 2018; Edoamodu and Nwodo 2022; Othman and Flaifil 2025; Bonnet et al. 2025). Laccases are naturally produced by a variety of microorganisms such as fungi, bacteria and plants, where fungal laccases have historically dominated research due to their high redox potential. However, bacterial laccases have gained increasing interest owing to their operational advantages, including broad tolerance to temperature fluctutaions, extreme pH and harsh industrial conditions, are particularly promising for industrial applications (Benali et al. 2024; Bozoglu 2014; Rezaei et al. 2017).

Despite these advantages, deploying bacterial laccases at industrial scale remains a challenge. Limitations such as enzyme instability, reduced activity in harsh environmental matrices, and difficulties in achieving cost‐effective large‐scale production hinder their practical utility (Bonnet et al. 2025; Wang et al. 2022). Consequently, optimising enzyme activity and production parameters is critical to overcoming these hurdles. Among various bacterial strains, *Bacillus atrophaeus* has recently attracted attention for its metabolic robustness, environmental resilience and capability to produce oxidative enzymes, including laccase. Its non‐pathogenic nature and ability to thrive in contaminated environments make it an attractive candidate for biotechnological applications. *B. atrophaeus* , a Gram‐positive, endospore‐forming bacterium from the class *Bacilli* , is commonly found in soil. It is known for producing a characteristic brown pigment and exhibits catalase and hemolytic activity while testing negative for oxidase, hydrogwn sulphide and indole. Additionally, *B. atrophaeus* is non‐motile and is recognised as a bacterial species known to produce antimicrobial compounds, making it a valuable microbial resource for biomolecule production (Bafana et al. 2011; Oladoye et al. 2022; Wang et al. 2022).

In bioremediation, particularly dye decolourisation, laccases play a crucial role in the oxidative breakdown of complex dye structures. Dyes such as Congo red, burazol navy and burazol black are extensively used in textiles, paper and aquaculture industries due to their vibrant colours and stability. They are designed to resist degradation under light, heat and chemical exposure, which makes their removal from industrial effluents particularly difficult. Their persistence in the environment contributes to water pollution, negatively affecting aquatic ecosystems and potentially entering the food chain. Moreover, many synthetic dyes, including azo dyes like Congo red, are known for their potential toxicity and carcinogenicity, posing risks to both environmental and human health (Zabłocka‐Godlewska et al. 2018; Ali 2010; Bafana et al. 2011; Oladoye et al. 2022; Pandey et al. 2007; Chung 2016).

Addressing the environmental impact of these dyes requires effective and sustainable decolourisation strategies. Effective and sustainable methods for dye decolourisation are critical for mitigating their environmental impact. At this stage, enzymes are crucial because of their ability to accelerate biochemical reactions by reducing the activation energy required. Moreover, their suitability for large‐scale applications and the significant advantages they provide in industrial settings highlight their significance. One of the frequently used enzymes for synthetic dye decolourisation is the laccase enzyme (Othman and Flaifil 2025; Zabłocka‐Godlewska et al. 2018; Ali 2010).

Optimisation focuses on assessing variable effects to achieve desired outcomes. Although factorial designs are widely used, they can be resource‐intensive, and identifying optimal conditions helps mitigate these challenges. In recent years, research has increasingly focused on enhancing laccase activity through statistical optimisation strategies. Although traditional ‘one‐factor‐at‐a‐time’ (OFAT) approaches provide basic insight, they are inefficient and fail to capture interactive effects among multiple parameters. In contrast, statistical methods such as response surface methodology (RSM) offer a powerful alternative by enabling the identification of optimal conditions through fewer experimental runs and revealing complex interactions between factors (Maniyam et al. 2024; Sarabia et al. 2020).

RSM is a statistical and mathematical technique introduced by Box and Wilson in 1951 to facilitate experimental optimisation. RSM is particularly useful for modelling and analysing problems in which a response of interest is influenced by multiple variables. It enables researchers to gain valuable insights with a limited number of experimental trials by predicting outcomes at untested points, thereby reducing uncertainty and improving efficiency (Robinson 2015; Cevik 2017; Mongomery 2001). RSM typically employs polynomial regression models, most often of second order (quadratic), to approximate the relationship between the response variable and the independent factors. Second‐order models are especially useful for representing complex relationships between factors and responses (Sella et al. 2014). Among various RSM designs, the central composite design (CCD) is widely used, particularly when studying continuous factors. It efficiently estimates both first‐ and second‐order terms, making it suitable for modelling and optimisation tasks. Other RSM designs include factorial, fractional factorial and Box–Behnken designs, which vary in complexity and application (Enez 2019).

The application of RSM to microbial enzyme systems has shown significant promise in improving enzyme yield, activity and stability. For example, a study demonstrated enhanced decolourisation of crystal violet by *Rhodococcus pyridinivorans* using optimised parameters identified via RSM (Maniyam et al. 2024). Similarly, other studies reported notable improvements in dye removal efficiencies by optimising laccase activity from *Coriolopsis gallica* and other fungal sources using mediator systems and statistical modelling (Zouari‐Mechichi et al. 2024; Ben Ayed et al. 2022). Despite the success of fungal systems, bacterial systems still lag in development, highlighting the need for further investigation.

In this study, we investigate *B. atrophaeus* , isolated from industrial wastewater, as a novel bacterial source of laccase. We focus on optimising its laccase activity using RSM, targeting key physicochemical and nutritional parameters such as pH, temperature, copper sulphate concentration, carbon and nitrogen sources, inoculum size and incubation time. The aim is to enhance laccase activity under laboratory conditions, with dye decolourisation of Congo red used as a functional validation example. Although dye decolourisation is included as a proof of concept, the central aim of this work is to advance the biotechnological application of bacterial laccases through effective enzyme optimisation, ultimately contributing to the development of more robust and scalable solutions for environmental remediation.

## 2 Material and Methods

### 2.1 Chemicals

Syringaldazyne (SGZ), guaiacol and 2,2‐Azino‐bis (3‐ethylbenzthiozoline‐6‐sulfonic acid) (ABTS) were purchased from Merck (Darmstadt, Germany). Congo red (sodium salt of benzidinediazo‐bis‐1‐naphthyl‐amine‐4‐sulfonic acid, C_32_H_22_N_6_Na_2_O_6_S_2_), burazol black (sodium salt of a bisazo compound, C_26_H_22_N_6_Na_2_O_8_S_2_) and burazol navy (sodium salt of a monoazo compound, C_23_H_16_N_4_Na_2_O_8_S_2_) were purchased from BURBOYA textile company in Bursa/Turkiye. Nutrient broth (NB) and nutrient agar (NA) medium were purchased from Merck (Darmstadt, Germany).

### 2.2 Microorganism and Laccase Source Preparation

Extracellular laccase‐producing *B. atrophaeus* was isolated from biological treatment sludge of the paper industry and identified based on 16S rRNA gene sequencing using A.B.T. 2X HS‐PCR MasterMix (with BlueDye) (P02‐02‐01, Turkey) device, branded as A.B.T., and a phylogenetic tree was constructed using MEGA version 4.0. Then, the presence of laccase enzyme was performed as indicated previously (Oztat et al. 2024).

*B. atrophaeus* was cultured following the McFarland 0.5 standard using the submerged culture method in 100 mL of NB at 37°C with continuous agitation at 150 rpm (Jeio Tech–IST, Turkiye) for 24 h. Afterwards, it was centrifuged (Hettich–Universal 320R, Germany) at 2200 × g at 4°C for 20 min, and the supernatant was stored at 4°C as an enzyme source (Kesebir 2020).

### 2.3 Laccase Assay

Laccase activity was measured using SGZ as a substrate (Khuri and Mukhopadhyay 2010). Three‐hundred microlitres of 0.5‐mM SGZ was added to 100 μL of enzyme source plus 600 μL of acetate buffer at pH 5.0. As a control, 700 μL of acetate buffer and 300 μL of SGZ were used, and oxidation was measured at 525 nm (*ε* = 65,000 mol^−1^ cm^−1^). One unit (U) of laccase was defined as the amount of enzyme required to convert 1 mmol substrate per minute under standard assay conditions (Kesebir 2020). Experiments were conducted with three biological and technical replicates.

### 2.4 Optimisation Studies

The medium and culture conditions were systematically optimised to enhance the laccase activity of *B. atrophaeus* . This optimisation aimed to achieve greater efficiency and produce outputs at reduced cost and energy consumption. Consequently, significant efforts were dedicated to refining both the culture conditions and the composition of the growth media. For the optimisation studies, minimal salt medium (MSM) was employed, comprising 0.5% carbon source, 0.5% nitrogen source, 7.8 g/L Na_2_HPO_4_·2H_2_O, 6.8 g/L KH_2_PO_4_, 0.2 g/L MgSO_4_, 0.05 g/L FeSO_4_, 0.05 g/L Ca(NO_3_)·4H_2_O and 0.08 g/L NaNO_3_.

#### 2.4.1 Traditional Method

The ‘OFAT’ approach was applied as a traditional method. To find the optimum medium and culture conditions; 5 different carbon sources (glucose, fructose, sodium acetate, carboxymethyl cellulose (CMC), xylose), 4 different nitrogen sources (KNO_3_, yeast extract, peptone from soybean, bacteriological peptone), 7 different incubation times (12, 24, 36, 48, 60, 72, 96 h), 7 different pH values (5.0, 6.0, 7.0, 8.0, 9.0, 10.0, 11.0), 4 different temperature (17°C, 27°C, 37°C, 47°C) and finally 3 different concentration of CuSO_4_ (0.5, 1, 1.5 mM) were studied respectively.

According to the results obtained from this study, the variables and their levels to be applied in the statistical method were determined. Experiments were conducted with three technical and biological replicates.

#### 2.4.2 Statistical Method‐RSM

The variables and levels selected to be applied in the response surface method are given in Table 1. Although determining the variables and levels, the results obtained from the traditional method and other literature studies were taken into consideration. The MINITAB software version 17.0 was used to perform the RSM studies.

**TABLE 1:** Selected variables and levels to be applied in RSM.

Isolate	Variable	Levels
*Bacillus atrophaeus*	pH	4
		6
		8
	Temperature	27°C
		37°C
		47°C
	CuSO_4_	0.5%
		1%
		1.5%
	Inoculum	0.50%
		1%
		1.50%
	Fructose	1 g/L
		3 g/L
		5 g/L
	Yeast extract	0.5 g/L
		1 g/L
		1.5 g/L

The sludge sample used in this study originated from an environment with ambient temperatures ranging between 32°C and 36°C. To ensure ecological relevance, experimental conditions were selected to align with this natural range. An initial OFAT analysis was conducted at four temperature levels: 17°C, 27°C, 37°C and 47°C, to evaluate the general temperature effect on laccase production. Based on the findings from this preliminary assessment, the RSM was applied using a narrowed temperature range of 27°C–47°C. This range was chosen to centre the optimisation design around the most promising temperature values identified during OFAT trials.

### 2.5 Enzyme Characterisation

Following the optimisation of medium and culture conditions, an enzyme characterisation study was conducted. This included evaluating the enzyme's activity with three substrates (SGZ, guaiacol, ABTS) at 0.5‐mM concentration across a range of pH values (4.0–11.0) using specific buffers (acetate, phosphate, Tris‐HCl, sodium‐carbonate). Additionally, substrate‐temperature optimisation was performed, testing the same substrates at temperatures from 20°C to 80°C. Finally, substrate concentration optimisation involved studying five different concentrations (0.5–2.5 mM) of ABTS.

### 2.6 Dye Decolourisation

The test solutions containing each dye were prepared by diluting a 1 mg/mL (1000 ppm) stock solution. The stock solution was prepared by dissolving the dye in distilled water and then filtering it through a 0.45‐μm membrane filter to ensure sterility. To achieve a final concentration of 50 ppm for each dye in the medium, the stock solution was diluted accordingly. Dyes were added to the medium post‐autoclaving to prevent degradation, ensuring that the medium itself was sterile while preserving the integrity of the dyes (Iscen et al. 2022; Ilhan et al. 2008). Experiments were conducted with three biological and technical replicates.

For the dye decolourisation; briefly, bacteria were streaked from a glycerol stock onto NA medium and incubated overnight at 37°C. The following day, a liquid culture was prepared in NB medium and incubated at 37°C with shaking at 150 rpm, overnight. Simultaneously, media containing synthetic dyes were prepared at a concentration of 50 ppm by autoclaving the NB medium, filtering the dyes through a 0.45‐μM membrane filter and adding them to the cooled NB medium. After the overnight incubation, a 1% inoculum of the bacterial culture (McFarland 0.5) was added to each dye solution in triplicate and incubated at 37°C with shaking at 150 rpm for up to 72 h. At 6, 24 and 72 h, the optical density (OD) was measured (Congo red at 350 nm, burazol black at 392 nm and burazol navy at 390.9 nm) by centrifuging samples at 7000 rpm for 15 min then reading the supernatant. The percentage decolourisation was calculated using the following equation:

(1) $$\%\text{decolourisation}=\left[\left(A_{0}-A_{t}\right)/A_{0}\right]\times 100$$

where *A*_0_ is the initial absorbance, and *A*_*t*_ is the absorbance at a given time.

The same process was applied under the optimal conditions for laccase activity obtained from RSM.

## 3 Results

### 3.1 Isolation, Laccase Enzyme Detection and Identification of Isolate

**FIGURE 1:**
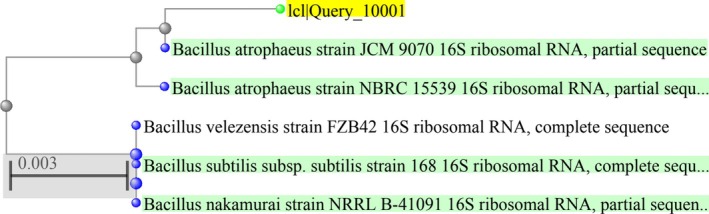
The phylogenetic tree of isolate and the type strains of closely related species based on partial 16S rRNA gene sequences.

Thirty‐two isolates were obtained by bacterial isolation from the biological treatment sludges of paper factories, and seven of them were considered to have laccase activity (based on the screening study with guaiacol), and the strain with the highest absorbance was selected for optimisation studies, as indicated previously (Oztat et al. 2024). As a result of morphological, physiological, biochemical and molecular tests, it was determined that the isolate was 99.71% similar to *B. atrophaeus* ( *Bacillus atrophaeus* strain JCM 9070 16S ribosomal RNA, partial sequence. Reference number: NR_024689.1) as given in Figure 1.

### 3.2 Optimisation With Traditional Method

**FIGURE 2:**
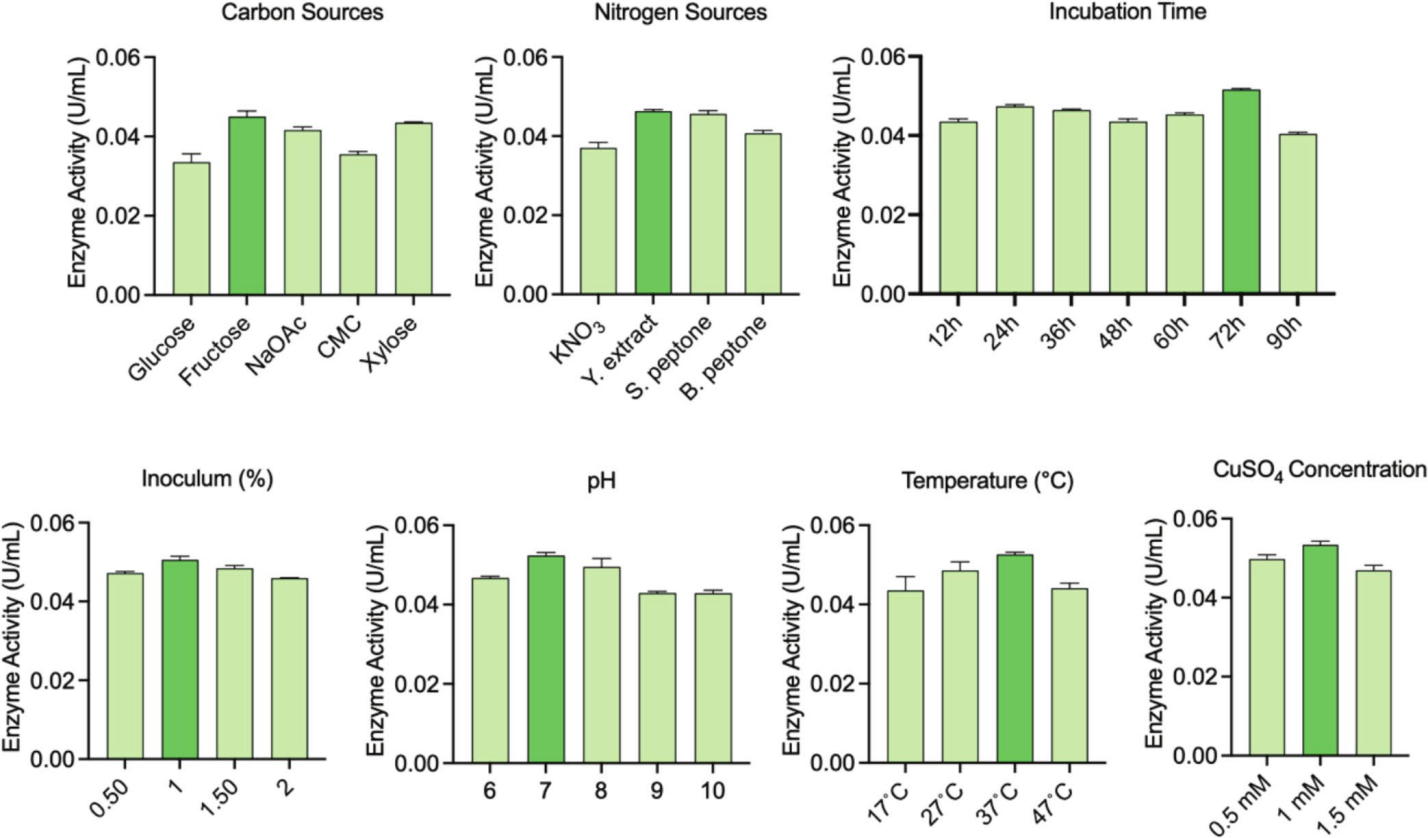
Enzyme activities of ‘one‐factor‐at‐a‐time’ methodology: carbon sources, nitrogen sources, incubation time, inoculum amount, pH, temperature and CuSO_4_ concentration, respectively.

The results of optimisation studies with ‘OFAT’ are given in Figure 2 (created using GraphPad Prism 9.5.1).

It was observed that isolate reached the highest enzyme activity with fructose as a carbon source and yeast extract as a nitrogen source; at 72 h of incubation with 1% inoculum, pH 7, at 37°C and 1‐mM concentration of CuSO_4_, respectively.

### 3.3 Response Surface Methodology

In the context of this study, the MINITAB program was used to analyse the CCD, a key component of RSM. The main effects, two‐way interaction effects, and quadratic effects were examined in this study. Variance analysis and *R* ^2^ results are provided in Table 2. As the variables with a *p* value below 0.05 are considered statistically significant, the experimental results for pH, temperature, CuSO_4_, fructose and yeast extract amount were considered statistically significant.

**TABLE 2:** Analysis of variance and *R* ^2^ results of the enzyme activity.

Analysis of variance			
Source	DF	Adj MS	*p*
**Model**	27	0.000034	0.000
**Linear**	6	0.000022	0.023
**pH**	1	0.000013	0.041
**Temperature**	1	0.000005	0.039
**CuSO**_4_	1	0.000003	0.028
Inoculum	1	0.000023	0.162
**Fructose**	1	0.000039	0.047
**Yeast extract**	1	0.000057	0.030
**Square**	6	0.000067	0.000
pH*pH	1	0.000193	0.000
Temperature*Temperature	1	0.000074	0.014
CuSO_4_*CuSO_4_	1	0.000044	0.056
Inoculum*Inoculum	1	0.000000	0.860
Fructose*Fructose	1	0.000035	0.088
Yeast*Yeast	1	0.000084	0.009
**2‐way interaction**	15	0.000026	0.014
pH*Temperature	1	0.000034	0.093
pH*CuSO_4_	1	0.000003	0.589
pH*Inoculum	1	0.000003	0.634
pH*Fructose	1	0.000150	0.001
pH*Yeast	1	0.000004	0.551
Temperature*CuSO_4_	1	0.000016	0.243
Temperature*Inoculum	1	0.000025	0.150
Temperature*Fructose	1	0.000002	0.705
Temperature*Yeast	1	0.000057	0.030
CuSO_4_*Inoculum	1	0.000009	0.382
CuSO_4_*Fructose	1	0.000037	0.078
CuSO_4_*Yeast	1	0.000000	0.949
Inoculum*Fructose	1	0.000008	0.422
Inoculum*Yeast	1	0.000029	0.120
Fructose*Yeast	1	0.000005	0.502
**Error**	62	0.000012	—
**Lack‐of‐fit**	48	0.000012	0.527
**Total**	89	0.001641	—
***R*** ^**2**^	**76.25%**		
***R*** ^**2**^ **(adj)**	**67.2%**		

*Note:* Bold text presented are statistically significant values. Abbreviations: Adj MS, adjusted mean square; DF, degrees of freedom.

When examining the model, it is observed that it accounts for enzyme production by a percentage of 76.25. However, upon addressing the shortcomings within the model, the rate at which this model represents enzyme activity is projected to be 67.20%.

The model developed from the analysis conducted is as follows in Equation (2).

(2) $$\begin{array}{c} \text{Enzyme activity}=0.0232-0.02725\;\mathrm{pH}+0.00455\;\text{Temperature}-0.0333\;{\text{CuSO}}_{4}-0.0070\;\text{Inoculum}\\ \;+0.00327\;\text{Fructose}+0.0581\;\text{Yeast}+0.002247\;{\mathrm{pH}}^{*}\mathrm{pH}-0.000056\;{\text{Temperature}}^{*}\text{Temperature}\\ \;+0.01716\;{{\text{CuSO}}_{4}}^{*}{\text{CuSO}}_{4}+0.00156\;{\text{Inoculum}}^{*}\text{Inoculum}-0.000953\;{\text{Fructose}}^{*}\text{Fructose}-0.02364\;{\text{Yeast}}^{*}\text{Yeast}\\ \;-0.000036\;{\mathrm{pH}}^{*}\text{Temperature}+0.000233\;{\mathrm{pH}}^{*}{\text{CuSO}}_{4}+0.000204\;{\mathrm{pH}}^{*}\text{Inoculum}+0.000385\;{\mathrm{pH}}^{*}\text{Fructose}\\ \;+0.000257\;{\mathrm{pH}}^{*}\text{Yeast}-0.000101\;{\text{Temperature}}^{*}{\text{CuSO}}_{4}+0.000125\;{\text{Temperature}}^{*}\text{Inoculum}-0.000008\;{\text{Temperature}}^{*}\text{Fructose}\\ \;-0.000191\;{\text{Temperature}}^{*}\text{Yeast}-0.00151\;{{\text{CuSO}}_{4}}^{*}\text{Inoculum}+0.000768\;{{\text{CuSO}}_{4}}^{*}\text{Fructose}+0.00011\;{{\text{CuSO}}_{4}}^{*}\text{Yeast}\\ \;+0.000346\;{\text{Inoculum}}^{*}\text{Fructose}-0.00270\;{\text{Inoculum}}^{*}\text{Yeast}-0.000289\;{\text{Fructose}}^{*}\text{Yeast} \end{array}$$

Per the formulation defining enzyme activity, the fixed coefficient is set at 0.0232. Upon elevating the pH value by a single unit, it introduces a reduction of −0.02725 into the model. Similarly, an increment of one unit in temperature imparts a positive addition of +0.00455 to the model. Upon meticulous examination, it was ascertained that the interaction effect between pH and temperature yields a contribution of −0.000036 to the model upon a unitary increase in either parameter. This pattern persists consistently across various factors under examination.

**FIGURE 3:**
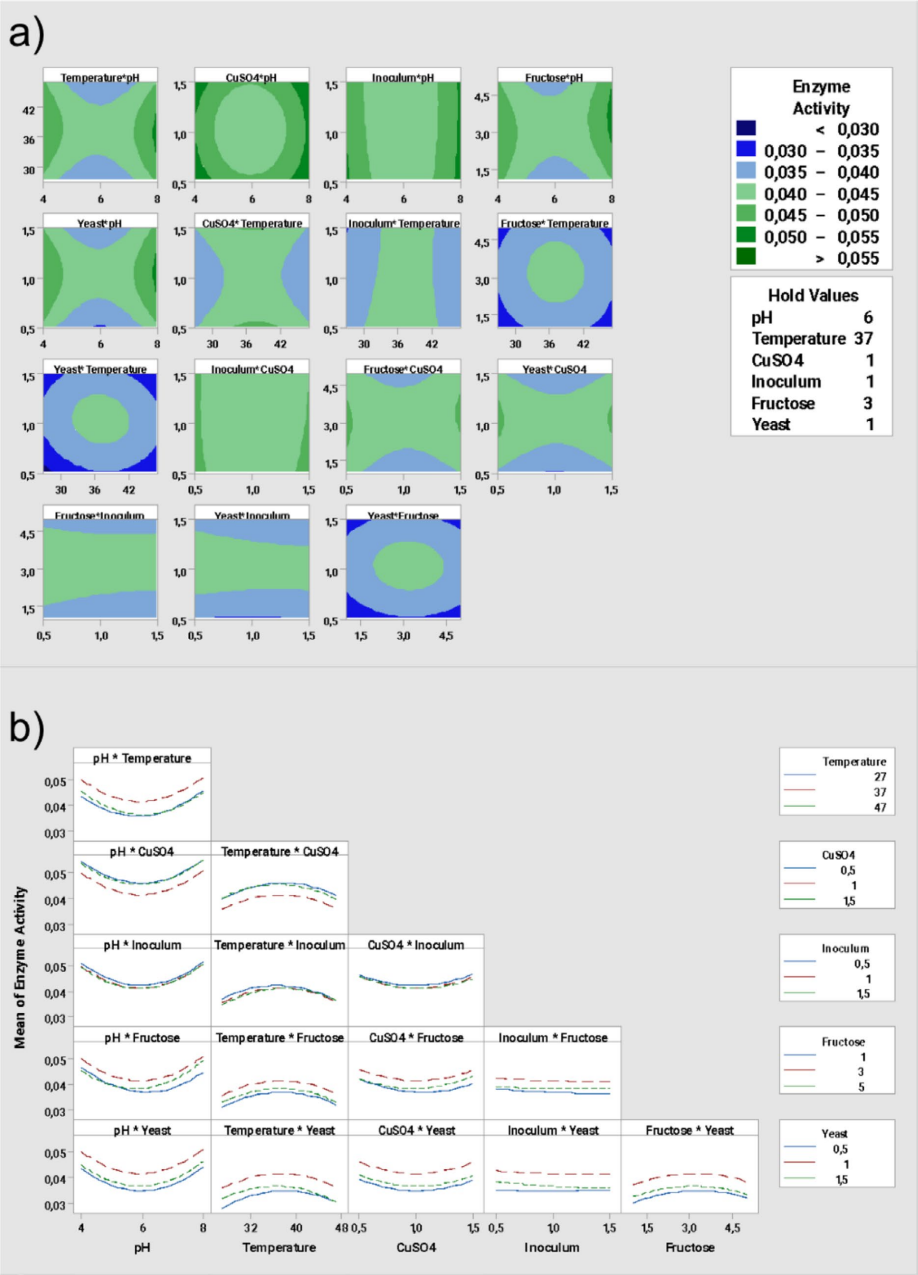
(a) Contour plots of enzyme activity. (b) Interaction plot for enzyme activity.

Contour plots and interaction plots of enzyme activity of the effects of binary interactions on enzyme activity are given below in Figure 3.

Figure 3a shows the effect of different factors on enzyme activity through binary combinations. Darkening shades of green in the contour graph indicate heightened enzyme activity. Considering this insight, the examination of the temperature–pH graph reveals that optimal enzyme activity is achieved at approximately 37°C, and the pH is 8; upon analysing the contour graph depicting the CuSO_4_‐fructose, it becomes evident that the enzyme activity reaches its peak when the CuSO_4_ concentration is approximately 1.5%, and the fructose concentration hovers around 3 g/L. In the context of the temperature–yeast extract, it is observed that the enzyme activity reaches its maximum when the temperature is approximately 37°C, and the yeast extract concentration is around 1 g/L. In the temperature–yeast extract, it can be observed that the enzyme activity reaches its maximum when the temperature is around 36°C, and the yeast extract concentration is approximately 1 g/L. In parallel, Figure 3b shows the fitted means of binary combinations; on the examination of fructose‐ yeast extract graph it is seen that enzyme activity reached its peak when the yeast extract 1 g/L, and fructose is 3 g/L in the culture medium.

**FIGURE 4:**
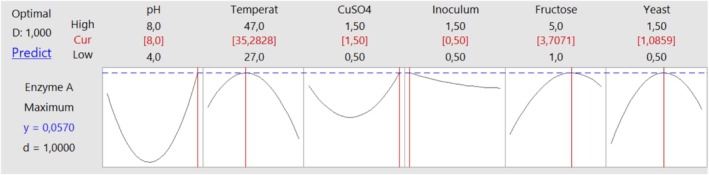
Graph illustrating the levels of variables required to achieve maximum enzyme activity from *B. atrophaeus* .

Figure 4 reveals the specific levels at which variables should be set to attain maximum laccase enzyme production from the *B. atrophaeus* .

Accordingly, to achieve the highest laccase enzyme activity, 0.057 U/mL, it is necessary to maintain a pH level of 8, a temperature of 35.28°C, a CuSO_4_ concentration of 1.5%, an inoculum quantity of 0.5 g/L, a fructose concentration of 3.7 g/L and a yeast extract concentration of 1.08 g/L.

### 3.4 Enzyme Characterisation

**FIGURE 5:**
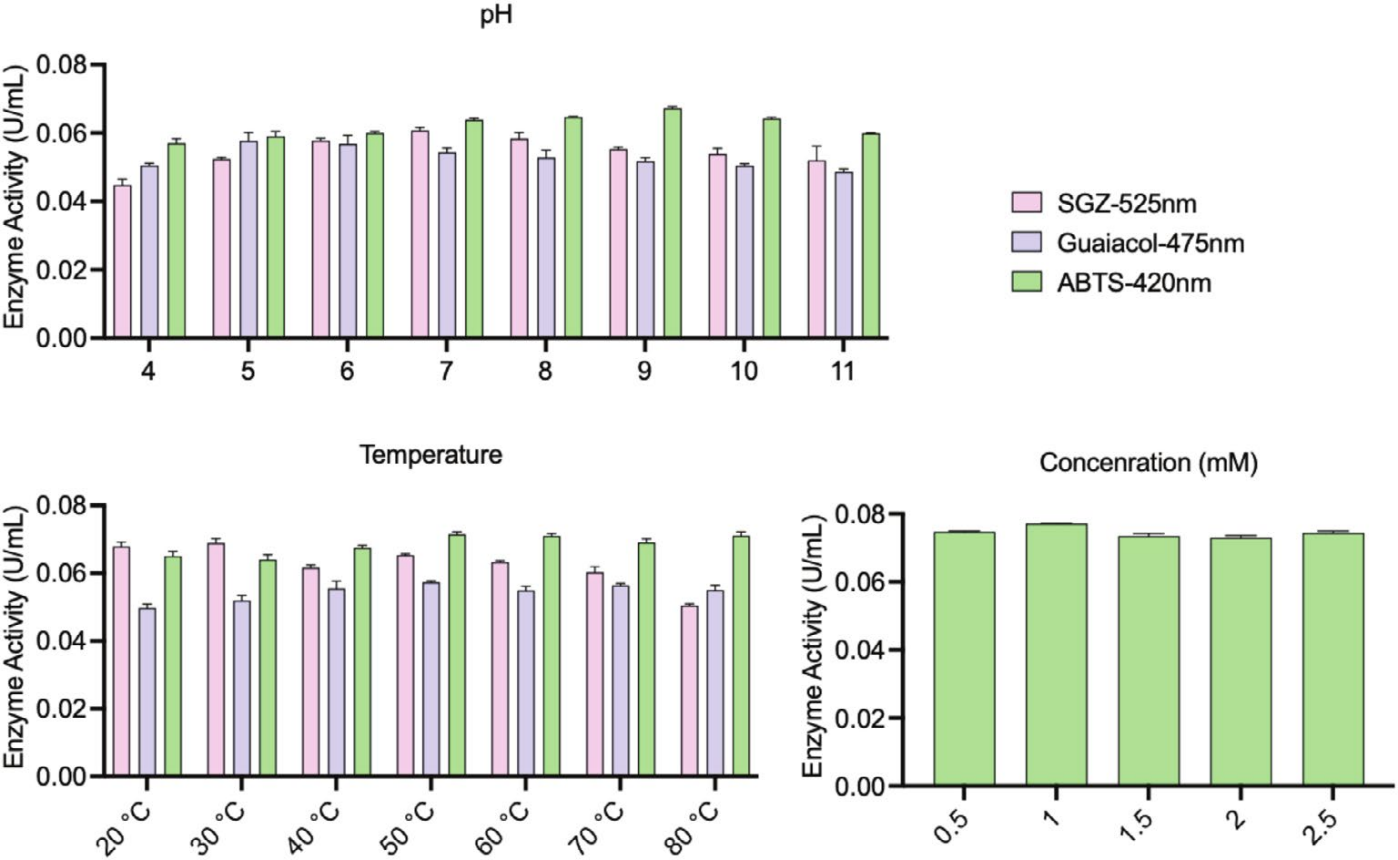
Enzyme's characteristics: substrate–pH optimisation, substrate–temperature optimisation and substrate concentration.

After optimising the medium and culture conditions, the enzyme characteristics were evaluated by examining substrate type, pH, temperature and substrate concentration effects on enzyme activity. Results are shown in Figure 5 (created using GraphPad Prism 9.5.1). The study tested eight pH values (4.0–11.0), seven temperatures (20°C–80°C), and three substrates (SGZ, guaiacol, ABTS). Maximum activity was achieved at pH 9.0 and 50°C with ABTS. Five ABTS concentrations (0.5–2.5 mM) were tested, with 1 mM found optimal. At the end, the results showed a 2.51‐fold increase in *B. atrophaues* laccase activity, reaching 0.0771 ± 0.0011 SD U/mL, compared to the initial activity, 0.0307 ± 0.0024 SD U/mL, measured before any optimisation process.

### 3.5 Decolourisation Efficiency

**FIGURE 6:**
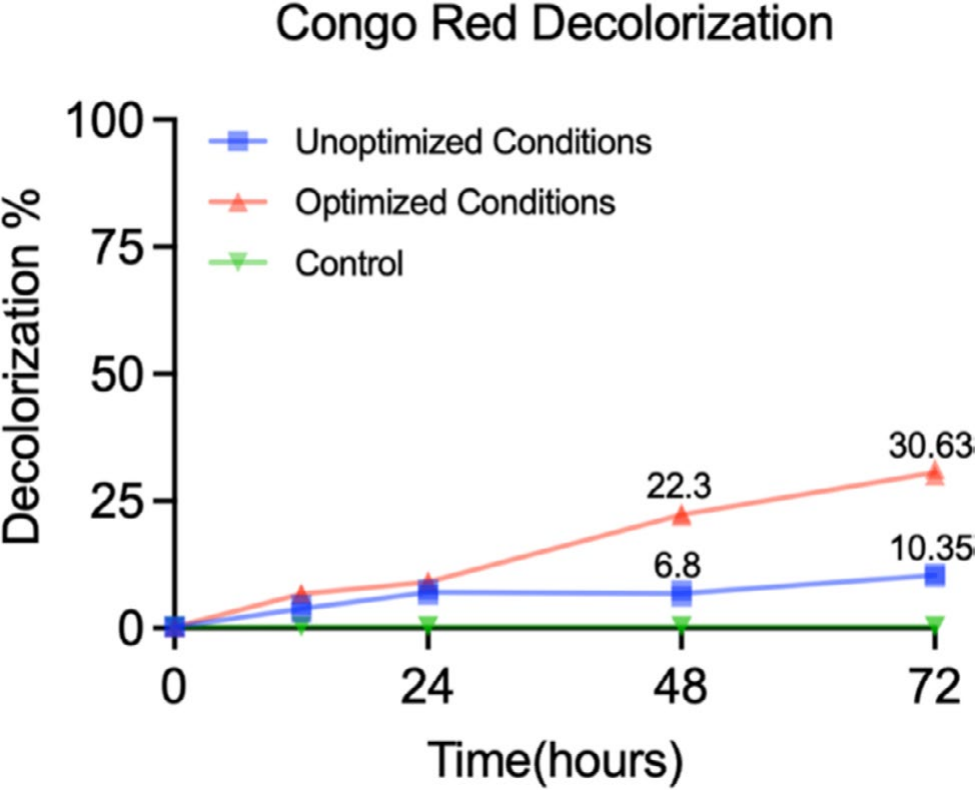
Comparison of Congo red decolourisation (%) under pre‐optimised and optimised conditions.

The decolourisation efficiency of *B. atrophaeus* was evaluated for Congo red, burazol black and burazol navy dyes under optimised and pre‐optimised conditions. Congo red, a carcinogenic and cytotoxic azo dye widely used in fertilisers, pesticides, optical films, highlighters and textiles, demonstrated valuable degradation. As illustrated in Figure 6 (generated using GraphPad Prism 9.5.1), the decolourisation of Congo red exhibited a 2.95‐fold increase compared to the pre‐optimised conditions after 72 h. In contrast, no decolourisation was observed for burazol black and burazol navy dyes under both pre‐optimised and optimised conditions.

## 4 Discussion

In recent years, the use and development of microbial enzymes in industrial applications has grown significantly, driven by the urgent demand for sustainable, cost‐effective and environmentally benign technologies. Among these biocatalysts, laccases (EC 1.10.3.2), a group of multicopper oxidases, have drawn increasing attention due to their ability to oxidise a wide range of phenolic and non‐phenolic compounds while utilising molecular oxygen and producing water as the sole byproduct (Othman and Flaifil 2025; Bonnet et al. 2025). The scalability and versatility of microbial laccases, particularly those produced by bacteria, present a strategic advantage over fungal counterparts due to bacteria's rapid growth rates, ease of genetic manipulation and ability to function under harsh industrial conditions (Maniyam et al. 2024; Benali et al. 2024).

The current study focused on optimising laccase activity in a locally isolated strain of *B. atrophaeus* , isolated from paper mill wastewater sludge, a niche environment that may select for strains capable of tolerating oxidative and xenobiotic stress. The ability of *B. atrophaeus* to produce laccase was validated and optimised using a two‐stage approach: initial screening via the OFAT method followed by a more robust multivariate optimisation using RSM. This approach enabled the systematic tuning of physicochemical parameters such as pH, temperature, CuSO_4_ concentration and nutrient levels to maximise enzyme output efficiently.

The isolation of *B. atrophaeus* from the biological treatment sludge of the paper industry, conducted within a temperature range of 32°C–36°C, highlighted the compatibility of the optimisation results with the environmental conditions during isolation. Notably, the observed optimum laccase activity at 35.28°C and slightly alkaline pH (8.0) reflects the thermotolerant and alkaliphilic traits typical of many industrial *Bacillus* species. These findings are consistent with reports highlighting the enhanced stability and catalytic efficiency of bacterial laccases under moderate thermal and non‐acidic conditions (Zouari‐Mechichi et al. 2024; Ben Ayed et al. 2022). The congruence between isolation and optimisation conditions supports the ecological adaptability and potential industrial applicability of *B. atrophaeus* as a robust laccase producer.

One of the most significant parameters in laccase regulation was CuSO_4_, serving not only as a cofactor but also as an inducer of laccase gene expression via copper‐responsive transcriptional regulators (Bonnet et al. 2025; Khaled et al. 2022; Deepa et al. 2020). This dual role is primarily mediated through copper‐responsive regulatory elements such as ACE1‐like transcription factors, which activate laccase expression in response to extracellular copper levels (Hernández‐Monjaraz et al. 2018; Khaled et al. 2022; Deepa et al. 2020). The optimisation study confirmed that the reasonable addition of CuSO_4_ significantly enhanced laccase activity. During RSM studies, the CuSO_4_ concentration was set at 1 mM, with only its quantity being varied, further corroborating the findings of the traditional optimisation study. The significant enhancement of laccase activity upon CuSO_4_ addition corroborates earlier reports that modest copper supplementation can dramatically increase enzymatic yield without exerting toxicity. In fungal systems such as *Pleurotus ostreatus* , the introduction of 1‐mM CuSO_4_ led to an 8‐fold enhancement in laccase activity without any noticeable negative impact on growth (Baldrian and Gabriel 2002). Although this study was conducted on fungi rather than bacteria, the stimulatory effect of copper on laccase production appears to be consistent across microbial groups. Variations in substrates and buffers used during spectrophotometric measurements of laccase activity led to differences in the results, underscoring the need for researchers to be aware of this variability in future studies and consider its optimisation.

In the enzyme characterisation assays, among the substrates tested (SGZ, guaiacol, ABTS), ABTS demonstrated the highest laccase activity. This observation aligns with previous studies where ABTS was identified as a preferred substrate for laccases due to its higher redox potential and solubility, facilitating efficient electron transfer during oxidation reactions (Oztat et al. 2024; Dai et al. 2021). Overall, the observed substrate preference and optimal conditions for *B. atrophaeus* laccase suggest its potential applicability in industrial processes that operate under alkaline and moderately high‐temperature conditions.

Following optimisation, the enzyme was tested for its ability to decolourise structurally complex synthetic dyes. Congo red, a model azo dye, exhibited a 2.95‐fold increase in decolourisation efficiency under optimised laccase conditions after 72 h. The success of Congo red degradation confirms the catalytic potential of the enzyme for breaking down azo bonds and aromatic rings, as reported in comparable studies (Maniyam et al. 2024; Benali et al. 2024). In contrast, burazol black and burazol navy were not effectively decolourised, which may be attributed to their sulfonic substituents and higher molecular complexity that hinder enzymatic access or oxidation, a limitation also observed in other studies when working with recalcitrant polyazo dyes (Zouari‐Mechichi et al. 2024). Importantly, the study reinforces the proof‐of‐concept role of dye decolourisation to validate enzymatic optimisation rather than being its primary objective. Although the enzyme shows promise, industrial application remains limited by issues of enzyme stability, reusability and economic viability. These challenges are well documented in the literature, particularly with bacterial laccases, which may exhibit lower turnover rates or substrate affinities than their fungal counterparts (Bonnet et al. 2025). Recent advancements in enzyme immobilisation, especially on carriers, such as calcium alginate and chitosan, have shown significant improvements in enzyme reusability and thermal tolerance, making them promising candidates for future enhancement (Othman and Flaifil 2025).

Moreover, addressing the resistance of specific dyes, such as burazol black and navy, may require co‐metabolic strategies, enzyme cocktails, or engineered laccase variants with broader substrate specificity. Recombinant expression systems, metabolic pathway engineering and directed evolution are promising avenues to tailor laccase systems for wider application (Bonnet et al. 2025; Ben Ayed et al. 2022).

## Author Contributions

**Kubra Kocak:** conceptualization (lead), data curation (equal), formal analysis (equal), investigation (equal), methodology (lead), software (equal), visualization (equal), writing – original draft (lead), writing – review and editing (lead). **Arzu Altin Yavuz:** conceptualization (equal), data curation (equal), formal analysis (equal), investigation (equal), software (lead), validation (equal), visualization (equal), writing – original draft (equal), writing – review and editing (equal). **Suleyman Berberler:** conceptualization (equal), investigation (equal), methodology (equal), writing – original draft (equal), writing – review and editing (equal). **Cansu Filik Iscen:** conceptualization (equal), data curation (equal), formal analysis (equal), funding acquisition (lead), investigation (lead), methodology (lead), project administration (lead), resources (lead), software (lead), supervision (lead), validation (lead), visualization (lead), writing – original draft (lead), writing – review and editing (lead).

## Conflicts of Interest

The authors declare no conflicts of interest.

## Data Availability

The datasets generated during and/or analysed during the current study are available from the corresponding author on reasonable request.

## References

[emi470138-bib-0001] Ali, H. 2010. “Biodegradation of Synthetic Dyes—A Review.” Water, Air, & Soil Pollution 213: 251–273.

[emi470138-bib-0002] Altıntas, B. 2011. “Removal of Textile Dyes From Wastewater Using Laccase Enzyme.” *Master's Degree Thesis*. 15–23.

[emi470138-bib-0003] Bafana, A. , S. S. Devi , and T. Chakrabarti . 2011. “Azo Dyes: Past, Present and the Future.” Environmental Reviews 19: 350–371.

[emi470138-bib-0004] Baldrian, P. , and J. Gabriel . 2002. “Copper and Cadmium Increase Laccase Activity in *Pleurotus ostreatus* .” FEMS Microbiology Letters 206, no. 1: 69–74. 11786259 10.1111/j.1574-6968.2002.tb10988.x

[emi470138-bib-0005] Ben Ayed, A. , B. Hadrich , G. Sciara , et al. 2022. “Optimization of the Decolorization of the Reactive Black 5 by a Laccase‐Like Active Cell‐Free Supernatant From *Coriolopsis gallica* .” Microorganisms 10, no. 6: 1137. 10.3390/microorganisms10061137. 35744655 PMC9227205

[emi470138-bib-0006] Benali, J. , I. Ben Atitallah , B. Ghariani , T. Mechichi , B. Hadrich , and H. Zouari‐Mechichi . 2024. “Optimized Decolorization of Two Poly Azo Dyes Sirius Red and Sirius Blue Using Laccase‐Mediator System.” 3 Biotech 14: 93. 10.1007/s13205-024-03937-4. PMC10907334 38433848

[emi470138-bib-0007] Bonnet, O. , T. Fa'aui , I. K. Leung , S. Yi , and W. Q. Zhuang . 2025. “Challenges and Applications of Laccase in Bioremediation.” In Laccase and Polyphenol Oxidase, 153–185. Elsevier.

[emi470138-bib-0008] Bozoglu, C. 2014. “Purification, Characterization, and Investigation of Industrial Usability of Laccase Enzyme From Thermophilic *Brevibacillus* sp. (Z1) Isolated From Ağrı Diyadin Hot Spring.” *Master's Degree Thesis*. 10–71.

[emi470138-bib-0009] Cevik, A. 2017. “ *Bacillus* sp. Determination of Optimum pH and Temperature Values of the Lakkaz Enzymin That Have Been Recognized.”

[emi470138-bib-0010] Chung, K. T. 2016. “Azo Dyes and Human Health: A Review.” Journal of Environmental Science and Health, Part C 34, no. 4: 233–261. 10.1080/10590501.2016.1236602 27635691

[emi470138-bib-0011] Dai, S. , Q. Yao , G. Yu , et al. 2021. “Biochemical Characterization of a Novel Bacterial Laccase and Improvement of Its Efficiency by Directed Evolution on Dye Degradation.” Frontiers in Microbiology 12: 633004. 34054745 10.3389/fmicb.2021.633004 PMC8149590

[emi470138-bib-0012] Deepa, T. , A. K. Gangwane , R. Z. Sayyed , H. P. Jadhav , and A. Mehjabeen . 2020. “Optimization and Scale‐Up of Laccase Production by *Bacillus* sp. BAB‐4151 Isolated From the Waste of the Soap Industry.” Environmental Sustainability 3, no. 4: 471–479.

[emi470138-bib-0013] Edoamodu, C. E. , and U. U. Nwodo . 2022. “Decolourization of Synthetic Dyes by Laccase Produced From *Bacillus* sp. NU2.” Biotechnology & Biotechnological Equipment 36, no. 1: 95–106.

[emi470138-bib-0014] Enez, B. 2019. “Isolation and Identification of *Bacillus atrophaeus* From Rhubarb Root Soil: Extraction and Characterization of α‐Amylase.” European Journal of Science and Technology 17: 736–743.

[emi470138-bib-0017] Hernández‐Monjaraz, W. S. , C. Caudillo‐Pérez , P. U. Salazar‐Sánchez , and K. L. Macías‐Sánchez . 2018. “Influence of Iron and Copper on the Activity of Laccases in *Fusarium oxysporum* f. sp. Lycopersici.” Brazilian Journal of Microbiology 49: 269–275. 10.1016/j.bjm.2018.06.002 PMC6328805 30145263

[emi470138-bib-0018] Ilhan, S. , C. F. Iscen , N. Caner , and I. Kiran . 2008. “Biosorption Potential of Dried Penicillium Restrictum for Reactive Orange 122: Isotherm, Kinetic and Thermodynamic Studies.” Journal of Chemical Technology & Biotechnology 83, no. 4: 569–575.

[emi470138-bib-0019] Iscen, C. F. , Ü. D. Gül , A. A. Yavuz , and S. İlhan . 2022. “Decolorization of Dye Solution Containing Remazol Black B by *Aspergillus niger* Isolated From Hypersaline Environment.” International Journal of Environmental Science and Technology 19, no. 12: 12497–12504.

[emi470138-bib-0020] Johannes, C. , and A. Majcherczyk . 2000. “Laccase Activity Tests and Laccase Inhibitors.” Journal of Biotechnology 78, no. 2: 193–199. 10725542 10.1016/s0168-1656(00)00208-x

[emi470138-bib-0021] Kesebir, A. 2020. “Purification of Laccase Enzyme From *B. licheniformis* O12 Bacteria, Recombinant Production From Bacteria DNA, Characterization, Biotechnological Applications.” *Dissertation*. University of Atatürk.

[emi470138-bib-0022] Khaled, J. M. , S. A. Alyahya , R. Govindan , et al. 2022. “Laccase Producing Bacteria Influenced the High Decolorization of Textile Azo Dyes With Advanced Study.” Environmental Research 207: 112211. 34656634 10.1016/j.envres.2021.112211

[emi470138-bib-0023] Khuri, A. I. , and S. Mukhopadhyay . 2010. “Response Surface Methodology.” Wiley Interdisciplinary Reviews: Computational Statistics 2, no. 2: 128–149.

[emi470138-bib-0024] Maniyam, M. N. , H. H. Azman , H. Abdullah , and N. S. Yaacob . 2024. “Response Surface Methodology as an Optimization Tool to Achieve an Effective Decolourization of Crystal Violet by the Malaysian *Rhodococcus pyridinivorans* Strain.” Biomass Conversion and Biorefinery 14, no. 10: 11023–11034.

[emi470138-bib-0025] Mongomery, D. C. 2001. Design and Analysis of Experiments. 5th ed. John Wiley & Sons, Inc.

[emi470138-bib-0027] Oladoye, P. O. , M. O. Bamigboye , O. D. Ogunbiyi , and M. T. Akano . 2022. “Toxicity and Decontamination Strategies of Congo Red Dye.” Groundwater for Sustainable Development 19: 100844.

[emi470138-bib-0028] Othman, A. M. , and A. G. Flaifil . 2025. “Characterization and Evaluation of the Immobilized Laccase Enzyme Potential in Dye Degradation via One Factor and Response Surface Methodology Approaches.” Scientific Reports 15, no. 1: 735. 39753629 10.1038/s41598-024-82310-0 PMC11699123

[emi470138-bib-0030] Oztat, K. , A. A. Yavuz , and C. F. Işçen . 2024. “Optimization Studies on Laccase Activity of *Proteus mirabilis* Isolated From Treatment Sludge of Textile Industry Factories: Optimization of Laccase Activity of *Proteus mirabilis* .” Brazilian Journal of Microbiology 55: 1231–1241. 38727921 10.1007/s42770-024-01350-w PMC11153439

[emi470138-bib-0031] Pandey, A. , P. Singh , and L. Iyengar . 2007. “Bacterial Decolorization and Degradation of Azo Dyes.” International Biodeterioration & Biodegradation 59, no. 2: 73–84.

[emi470138-bib-0032] Rezaei, S. , A. R. Shahverdi , and M. A. Faramarzi . 2017. “Isolation, One‐Step Affinity Purification, and Characterization of a Polyextremotolerant Laccase From the Halophilic Bacterium Aquisalibacillus Elongatus and Its Application in the Delignification of Sugar Beet Pulp.” Bioresource Technology 230: 67–75. 28161622 10.1016/j.biortech.2017.01.036

[emi470138-bib-0033] Robinson, P. K. 2015. “Enzymes: Principles and Biotechnological Applications.” Essays in Biochemistry 59: 1–41. 26504249 10.1042/bse0590001 PMC4692135

[emi470138-bib-0034] Rodriguez‐Couto, S. 2018. “Solid‐State Fermentation for Laccases Production and Their Applications.” In Current Developments in Biotechnology and Bioengineering, 211–234. Elsevier.

[emi470138-bib-0035] Sarabia, L. A. , M. C. Ortiz , and M. S. Sanchez . 2020. Response Surface Methodology, 287–326. Elsevier.

[emi470138-bib-0036] Sella, S. R. B. R. , L. Vandenberghe , and C. Soccol . 2014. “ *Bacillus atrophaeus* : Main Characteristics and Biotechnological Applications—A Review.” Critical Reviews in Biotechnology 35, no. 4: 533–545. 24963702 10.3109/07388551.2014.922915

[emi470138-bib-0039] Wang, L. , Y. Tan , S. Sun , et al. 2022. “Improving Degradation of Polycyclic Aromatic Hydrocarbons by *Bacillus atrophaeus* Laccase Fused With Vitreoscilla Hemoglobin and a Novel Strong Promoter Replacement.” Biology 11, no. 8: 1129. 36009756 10.3390/biology11081129 PMC9404780

[emi470138-bib-0040] Zabłocka‐Godlewska, E. , W. Przystaś , and E. Grabińska‐Sota . 2018. “Possibilities of Obtaining From Highly Polluted Environments: New Bacterial Strains With a Significant Decolorization Potential of Different Synthetic Dyes.” Water, Air, and Soil Pollution 229: 1–13. 10.1007/s11270-018-3829-7 PMC5962626 29861514

[emi470138-bib-0041] Zouari‐Mechichi, H. , J. Benali , A. H. Alessa , B. Hadrich , and T. Mechichi . 2024. “Efficient Decolorization of the Poly‐Azo Dye Sirius Grey by *Coriolopsis gallica* Laccase‐Mediator System: Process Optimization and Toxicity Assessment.” Molecules 29, no. 2: 477. 10.3390/molecules29020477. 38257390 PMC10819905

